# Mixed Adenoma and Well-Differentiated Neuroendocrine Tumor (MANET) of the Middle Ear

**DOI:** 10.1007/s12022-024-09811-6

**Published:** 2024-05-25

**Authors:** Ivan J. Stojanov, Sylvia L. Asa

**Affiliations:** 1 Department of Pathology, Robert J. Tomsich Pathology and Laboratory Medicine Institute, Cleveland Clinic, Cleveland, OH USA; 2 Department of Pathology, University Hospitals Cleveland Medical Center, Case Western Reserve University, Cleveland, OH USA

## Case History

A 45-year-old male presented with acute mastoiditis and facial nerve paralysis. Computerized tomography of the temporal bone demonstrated complete opacification of left mastoid air cells, near complete middle ear cavity opacification, and bony erosion of the petrous apex extending to the left geniculate ganglion and proximal facial nerve canal. There was associated tympanic membrane thickening and external auditory canal debris. The patient was referred for mastoidectomy at which point a biopsy was performed.

## What Is Your Diagnosis?

**Fig. 1:**
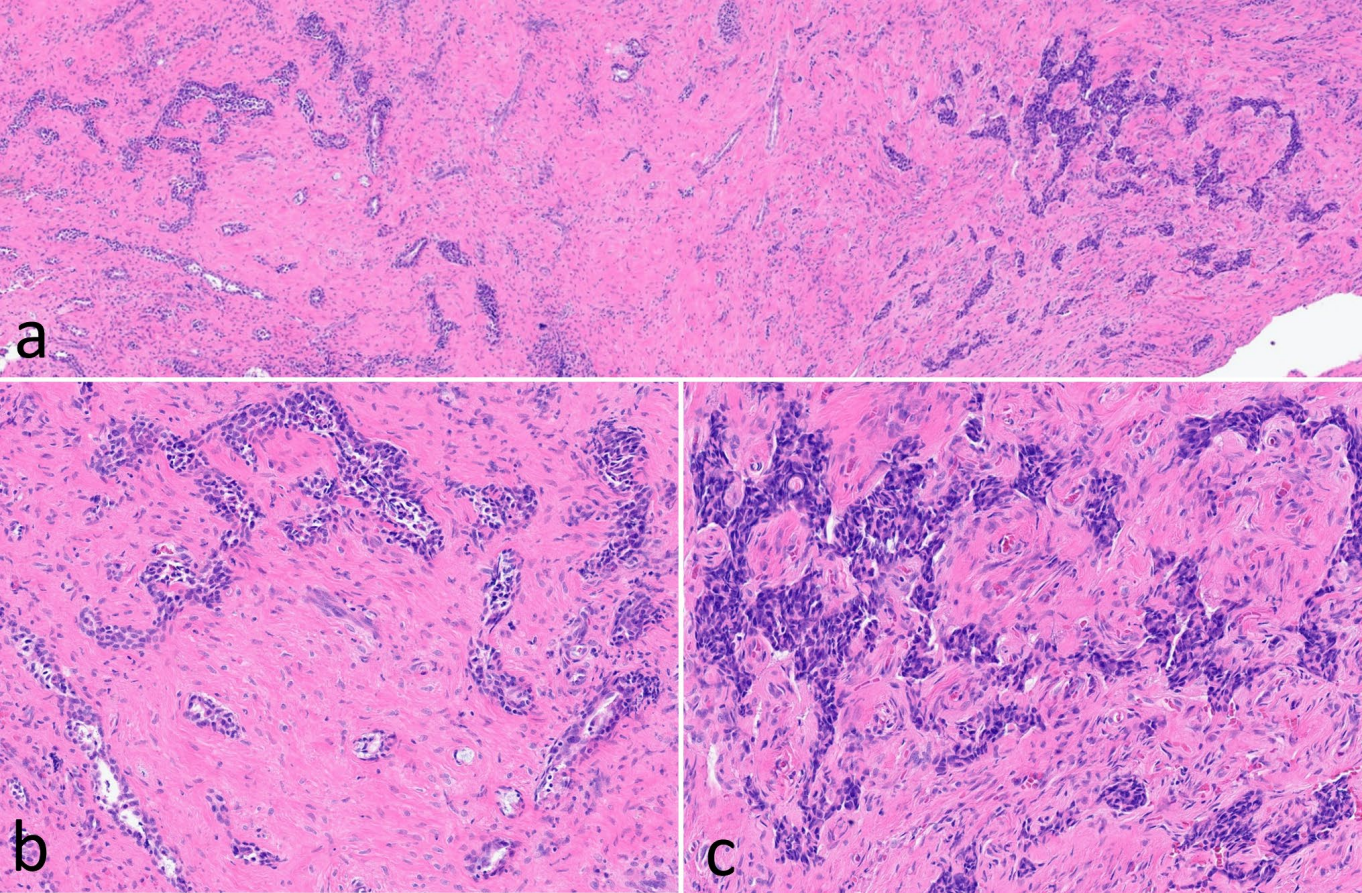
MANET presenting with distinctive non-neuroendocrine (left) and neuroendocrine (right) components (**a**); non-neuroendocrine component exhibits tubuloglandular growth (**b**), whereas neuroendocrine component exhibits corded to nested growth (**c**)

**Fig. 2:**
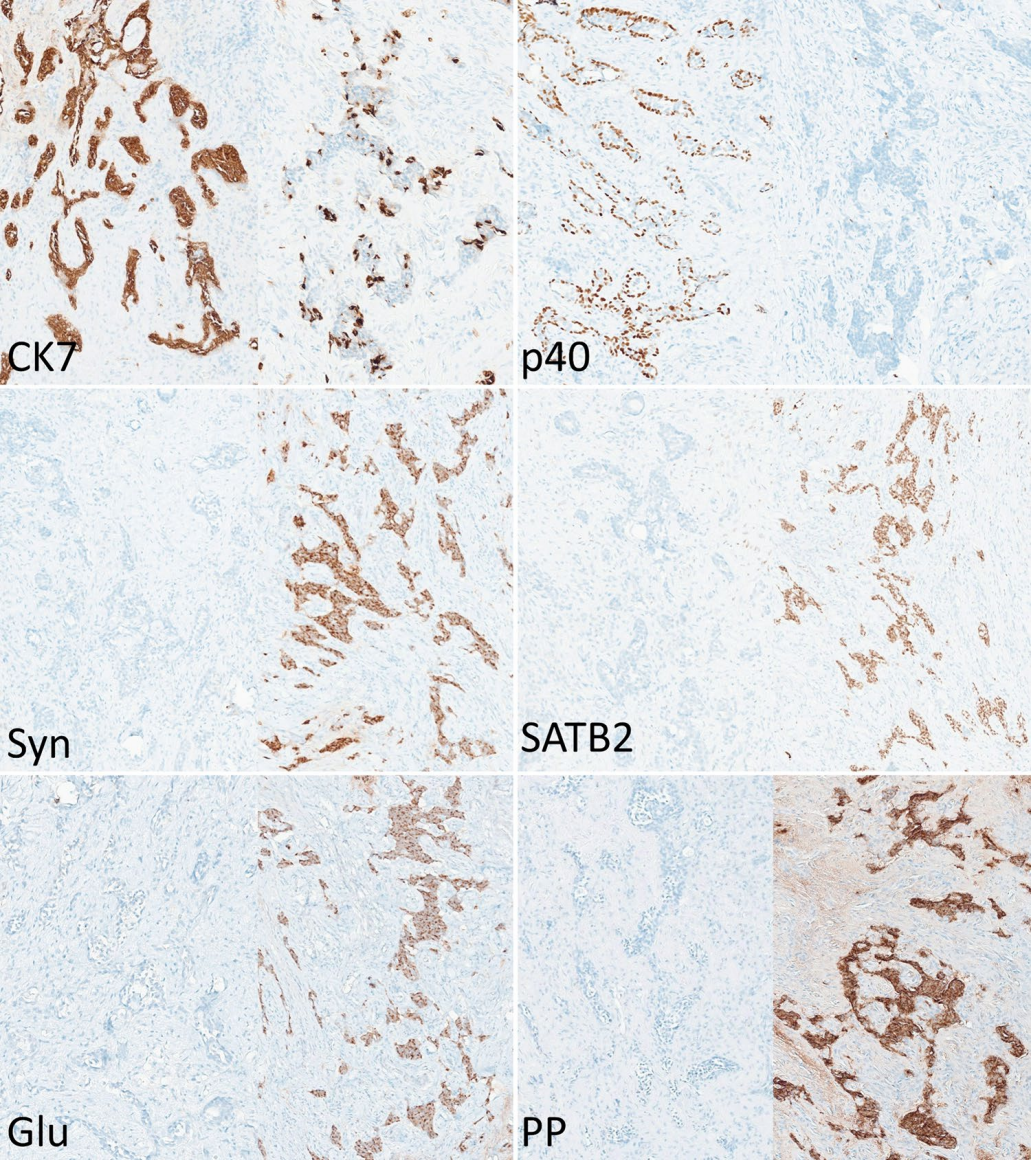
In all figure parts, adenoma component is on left and neuroendocrine component is on right. CK7 is strongly positive in adenoma component and highlights only scattered cells in neuroendocrine component. p40 highlights abluminal cells in adenoma component and is negative in neuroendocrine component. Synaptophysin (Syn), SATB2, glucagon (Glu), and pancreatic polypeptide (PP) are negative in adenoma component while positive in neuroendocrine component

See figure composites (Figs. 1 and 2).

## Diagnosis: Mixed Adenoma and Well-Differentiated Neuroendocrine Tumor (MANET)

Histologic evaluation identified a neoplasm with two distinct growth patterns, tubuloglandular and trabecular/cord-like, each comprising approximately half (~ 50%) of the tumor (Fig. 1). Tumor cells were hyperchromatic in both portions of the tumor, with more appreciable eosinophilic cytoplasm in areas of tubuloglandular growth. Intraneural invasion was present, but there was no appreciable mitotic activity (0/2 mm^2^), tumor necrosis, or poorly differentiated cytomorphology. Immunohistochemistry demonstrated complementary staining patterns in the different portions of the tumor, with the tubuloglandular portion positive for CK7 and p40 (in basal/abluminal cells) and the trabecular/cord-like portion positive for synaptophysin, chromogranin (weak), INSM1, SATB2, glucagon, pancreatic polypeptide, and SSTR2A (Fig. 2). Immunostains positive in one portion of the tumor were negative in the other, except for CK7 which was strong and diffuse in the non-neuroendocrine component and also showed scattered positivity in the neuroendocrine component; the focus of intraneural invasion demonstrated neuroendocrine differentiation by immunohistochemistry. All tumor cells were positive for AE1/AE3. Ki-67 labeled less than 3% of cells in the neuroendocrine component and approximately 5–10% in the non-neuroendocrine component. The histologic and immunohistochemical findings supported the diagnosis of mixed adenoma/well-differentiated L cell neuroendocrine tumor (MANET), an indolent subtype of mixed neuroendocrine and non-neuroendocrine neoplasm (MiNEN), of the middle ear. Unfortunately, the patient was lost to follow-up.

## Comment

MiNEN is a mixed epithelial neoplasm with combined neuroendocrine and non-neuroendocrine components. Each component is morphologically and immunohistochemically recognizable and comprises at least 30% of the tumor. MiNEN was first identified in the digestive system, where it has been best characterized, and has been subsequently observed to occur in virtually every organ system [1]. Occasionally, as in this case, both components of a MiNEN may be histologically indolent with no features of adenocarcinoma or neuroendocrine carcinoma. Such tumors are classified as “MANET,” mixed adenoma and well-differentiated neuroendocrine tumor, and these tumors have been shown to pursue an indolent course in the digestive system [2].

In the middle ear, the terminology middle ear neuroendocrine tumor (MeNET) has recently replaced that of middle ear adenoma on the basis of evidence of neuroendocrine differentiation and clinical behavior comparable to NETs occurring elsewhere [3]. Whether MiNEN of any sort can occur in the middle ear has been a matter of some debate. MeNETs are well-recognized to exhibit morphologic diversity in terms of patterns of growth, not only between different tumors but also within the same tumor, which has historically suggested divergent differentiation and resulted in a variety of names for these tumors including middle ear adenoma, carcinoid, neuroendocrine adenoma of the middle ear, and middle ear adenomatous neuroendocrine tumor. In an AFIP series of 48 cases, only 19% of tumors demonstrated a single growth pattern, and glandular-type growth was the most commonly identified of the various growth patterns [4]. However, recent immunohistochemical studies have shown generally uniform expression of INSM1, SATB2, ISL1, synaptophysin, and peptide hormones in MeNET, regardless of growth pattern and including in scattered tumor cells that express CK7, suggesting a single lineage in spite of morphologic heterogeneity [5]. Moreover, there is absence of myoepithelial differentiation in these tumors, including in areas of supposed tubuloglandular growth [5].

Given the morphologic diversity and rarity of MeNET, true middle ear mixed neuroendocrine and non-neuroendocrine neoplasms (MeMiNEN) are, at best, exceedingly uncommon and yet to be well-described immunohistochemically. In this case, we demonstrate a middle ear tumor with distinctive neuroendocrine and non-neuroendocrine components, in keeping with the definition of MiNEN. The neuroendocrine portion of the tumor was positive for neuroendocrine markers INSM1, synaptophysin, chromogranin, and SSTR2A, as well as the recently identified markers of L-cell differentiation SATB2, glucagon and pancreatic polypeptide, and lacked histologic features of neuroendocrine carcinoma. The tubuloglandular portion was positive for CK7 and p63 (in abluminal cells); similarly, histologic features of middle ear adenocarcinoma including architectural complexity with cystic-papillary growth were absent. The immunoprofile was complementary in that markers positive in one portion of the tumor were negative in the other, with the exception of scattered CK7 positivity in the neuroendocrine portion of the tumor, as has been previously described in MeNET.

In conclusion, this case demonstrates morphologically and immunohistochemically that true MiNEN may occur in the middle ear. As in the digestive system, middle ear MiNEN may consist of histologically low-grade adenoma and well-differentiated neuroendocrine tumor components, in which setting the tumor may be best referred to as MANET. Tubuloglandular-type growth alone does not support evidence of MiNEN as it has been well-described in conventional MeNET, but should prompt immunohistochemical workup in consideration of MeMiNEN.

## Data Availability

No datasets were generated or analysed during the current study.

## Declarations

Dr. Asa is a member of the Editorial Board of Endocrine Pathology.
